# The Ca^2+^-Activated K^+^ Channel KCa3.1 as a Potential New Target for the Prevention of Allograft Vasculopathy

**DOI:** 10.1371/journal.pone.0081006

**Published:** 2013-11-29

**Authors:** Yi-Je Chen, Jenny Lam, Clare R. Gregory, Sonja Schrepfer, Heike Wulff

**Affiliations:** 1 Department of Pharmacology, University of California, Davis, California, United States of America; 2 Department of Surgical and Radiological Sciences, School of Veterinary Medicine, University of California, Davis, California, United States of America; 3 University Heart Center Hamburg, Transplant and Stem Cell Immunobiology Lab, Hamburg, Germany; 4 Department of Cardiothoracic Surgery Stanford University School of Medicine, Stanford, California, United States of America; Sackler Medical School, Tel Aviv University, Israel

## Abstract

Allograft vasculopathy (AV) remains one of the major challenges to the long-term functioning of solid organ transplants. Although its exact pathogenesis remains unclear, AV is characterized by both fibromuscular proliferation and infiltration of CD4^+^ memory T cells. We here tested whether two experimental immunosuppressants targeting K^+^ channels might be useful for preventing AV. PAP-1 inhibits the voltage-gated Kv1.3 channel, which is overexpressed on CCR7^−^ memory T cells and we therefore hypothesize that it should suppress the memory T cell component of AV. Based on its previous efficacy in restenosis and kidney fibrosis we expected that the KCa3.1 blocker TRAM-34 would primarily affect smooth muscle and fibroblast proliferation and thus reduce intimal hyperplasia. Using immunohistochemistry we demonstrated the presence of Kv1.3 on infiltrating T cells and of KCa3.1 on lymphocytes as well as on proliferating neointimal smooth muscle cells in human vasculopathy samples and in a rat aorta transplant model developing chronic AV. Treatment of PVG rats receiving orthotopically transplanted aortas from ACI rats with TRAM-34 dose-dependently reduced aortic luminal occlusion, intimal hyperplasia, mononuclear cell infiltration and collagen deposition 120 days after transplantation. The Kv1.3 blocker PAP-1 in contrast did not reduce intima hyperplasia despite drastically reducing plasma IFN-γ levels and inhibiting lymphocyte infiltration. Our findings suggest that KCa3.1 channels play an important role in the pathogenesis of chronic AV and constitute an attractive target for the prevention of arteriopathy.

## Introduction

Allograft vasculopathy (AV), a concentric thickening of the arteries in transplanted hearts or kidneys ultimately leading to luminal obliteration and thus ischemic graft failure, remains one of the major challenges to the long term functioning of solid organ transplants [1]. AV, which is sometimes called transplant arteriosclerosis resembles atherosclerosis in many respects. In both diseases the endothelium is dysfunctional and damaged; fostering inflammation, increased intimal thickening, and eventually the development of medial smooth muscle cell degeneration, and adventitial fibrosis [2]. Histopathology in both conditions demonstrates the involvement of T cells, monocytes/macrophages, and proliferating vascular smooth muscle cells as well as fibrotic changes. However, in contrast to atherosclerotic plaques, which are typically eccentric, the fibromuscular proliferation characteristic of AV tends to be cirumferential and can affect both veins and arteries [3]. The exact pathogenesis of AV remains currently unclear but it seems to have both a fibroproliferative and a CD4^+^ T-cell mediated component and thus differs fundamentally from the CD8^+^ T cell response against class I transplantation antigens. Evidence for Peter Libby’s original hypothesis [4] that AV represents an ineffective delayed-type-hypersensitivity (DTH) response against donor endothelial cells and medial smooth muscle cells comes from observations that CD4^+^ T cells outnumber CD8s 2:1 in the neointima and adventitia of human coronary arteries with AV [5] and that the infiltrating cells are predominantly memory Th1 cells producing IFN-γ [1]. However, the fact that AV can even occur following ischemic injury in isografts [6] or in T-cell depleted hosts after a transient episode of rejection [7], suggests that once initiated, dedifferentiated smooth muscle cells of both donor and recipient origin as well as activated and injured endothelial cells participate in the ongoing vasculopathy ultimately leading to luminal obliteration. Unfortunately, most clinically used immunosuppressive regiments, while quite effective at preventing acute allograft rejection, fail to prevent AV and 50% of grafts will show significant arteriopathy within 5 years after transplantation, while 90% will be affected within 10 years [1].

The voltage-gated Kv1.3 and the calcium-activated KCa3.1 potassium channels constitute two promising new anti-inflammatory drug targets. Both channels play important roles in lymphocyte activation by regulating membrane potential and calcium signaling [8]. While Kv1.3 is predominantly expressed in T cells, B cells and macrophages and is up-regulated in CCR7^−^ effector memory T cells [9], [10], KCa3.1 is found on activated CCR7^+^ T cells, IgD^+^ B cells, and macrophages as well as on proliferating dedifferentiated vascular smooth muscle cells, vascular endothelium and fibroblasts (see [[8], [11], [12]]) for extensive reviews). Based on this expression pattern, Kv1.3 blockers are currently in Phase-1 clinical trials for multiple sclerosis [13] and psoriasis, while KCa3.1 blockers are being investigated for conditions such as asthma, restenosis disease, kidney fibrosis and atherosclerosis, which in addition to involving T cells and macrophages also have a vascular smooth muscle cell and fibroblast proliferative component [14]. We therefore hypothesized that PAP-1 and TRAM-34, small molecule blockers of Kv1.3 and KCa3.1 which were designed by our group 15,16, might be able to prevent the development of allograft vasculopathy. Our reasoning here was that PAP-1, which has been previously reported to effectively suppress DTH [15] and allergic contact dermatitis [17] (both memory T cell mediated conditions) as well as to prevent autoimmune diabetes in MHC class II-restricted diabetes-prone BB/W rats [10], would target the DTH-like T cell-mediated component in the pathogenesis of allograft vasculopathy. The KCa3.1 blocker TRAM-34 in contrast should primarily affect the smooth muscle and fibroproliferative component of the disease based on previous findings that the compound prevents restenosis following balloon angioplasty in rats [18] and pigs [19], kidney fibrosis in mice and rats [20], and atherosclerosis development in ApoE^−/−^ mice [21]. We are accordingly here investigating Kv1.3 and KCa3.1 expression in human vasculopathy samples and are testing TRAM-34 and PAP-1 in an orthotopic aorta transplant model in a rat strain combination which is known to develop slow, chronic vasculopathy [22].

## Materials and Methods

### Patients

Human studies were performed in accordance with the Declaration of Helsinki and were approved by the Board of Physicians Committee Hamburg. Patients providing tissue were treated in the Department of Cardiovascular Surgery at the University Heart Center Hamburg and gave their written informed consent to use part of their left-over vessels for experimental purposes. The Internal mammary artery (IMA) vessel was freshly obtained from a patient undergoing coronary artery bypass graft (CABG) surgery (>60 years) followed by fixation using 4% paraformaldehyde. Massive intimal hyperplasia was confirmed by histopathology. Human coronary arteries with atherosclerotic plaques: Human coronary artery samples from patients (n = 2) undergoing heart transplantation with preexisting disease or calcifications were harvested from the former heart of the patients and fixed for histopathology.

### Orthotopic aorta transplant

This study was approved by the University of California, Davis, Animal Use and Care Committee and conducted in accordance with the guidelines of Animal Use and Care of the National Institutes of Health and the University of California, Davis for survival surgery in rodents. A section of the thoracic aorta from PVG (allo-transplant) or ACI (iso-transplant) rats was orthotopically transplanted into the infrarenal abdominal aorta of ACI rats by end to end anastomoses according to the method of Mennander et al. [23]. Briefly, the donor rats were anesthetized using box induction with isoflurane (5% in medical air). The rats were then removed from the box and maintained on 0.5–1.5% isoflurane via a face mask. The rat was placed in dorsal recumbancy on a heating pad for a ventral celiotomy. The abdomen was clipped prepped with 0.5% chlorhexidine and rinsed with water. A fine spray of 0.5% chlorhexidine was placed over the surgical area prior to placing a sterile drape. A ventral celiotomy was performed to expose the caudal vena cava and injected 50 IU/kg of heparin into it. Following a 3 minute period, the infrarenal aorta and vena cava were severed and the rat was exsanguinated. The thorax is then entered and the thoracic aorta was harvested for transplantation, flushed with heparinized saline and stored in cold Ringer's solution. The recipient rats were anesthetized and prepared for surgery in the same manner as the donor rats. A ventral celiotomy was performed. The infrarenal aorta was isolated and clamped proximal and distal to the anastomosis site and cut and flushed with heparinized saline. The aortic allograft was sutured to the recipient aorta using 8–0 nylon in a running pattern. The clamps were released and the segment tested for patency. The linea was closed with 4–0 PDS in a simple continuous pattern and the subcuticular tissue was closed with 4–0 monocryl in a simple continuous pattern. The rats were recovered on circulating warm water pads and towels and had heat lamps focused on the recovery area. Postsurgical animals were monitored for signs of pain, distress, dehydration, weight loss and general well-being. Monitoring occurred every 8 hours during the first day after surgery and then daily for the next 119 days. Buprenorphine was given (5 µg/kg s.c. every 8 h for 24 h) for pain control.

After transplantation, all recipients received 5 mg/kg cyclosporine A (Neoral®, Novartis, Basel, Switzerland) for 7 days by oral gavage and were then randomly assigned to one of nine different treatment groups: miglyol vehicle i.p. (Neobee5, Spectrum, NJ, USA), TRAM-34 i.p at 10 mg/kg or 40 mg/kg (synthesized in Wulff laboratory as previously described [16]), sirolimus orally at 0.3 mg/kg or 1 mg/kg (Rapamune®, Wyeth, Münster, Germany), a combination of TRAM-34 (10 mg/kg i.p.) and sirolimus (0.3 mg/kg orally), PAP-1 i.p. at 40 mg/kg (synthesized in Wulff laboratory as described [15]), or isograft treated i.p. with miglyol vehicle. The details of the treatment plan are shown in Figure 3. Animals were scarified 120 days after transplantation with an overdose of isoflurane. One animal in the high-dose rapamycin group developed an infection and was euthanized with an overdose of isoflurane. No animals died during the study period. The transplanted aorta was collected and fixed in 10% formalin for 24 h, then embedded in paraffin and sectioned for histopathological examinations. Plasma was collected for determination of IFN-γ concentrations and drug levels and immediately frozen at –80°C pending analysis.

### Heterotopic heart transplant

All animal procedures were approved by the Animal Care and Use Committee of Stanford University and conducted in accordance with the guidelines of Animal Use and Care of the National Institutes of Health. Adult male (10 weeks old, 270 to 300 g) Lewis (RT1^1^) and Brown Norway (BN, RT1^a^) rats were purchased from Harlan Laboratories (Indianapolis, IN, USA) and hearts heterotopically transplanted to the abdominal great vessels from BN to Lewis rats as described [24], [25].

Donor rats were anesthetized with isoflurane, the abdomen and thorax clipped and prepared for aseptic surgery. The animals were then placed in dorsal recumbancy and a midline abdominal incision was made from the xiphoid to the pubis. The caudal vena cava was located and 0.5 ml of heparin (500 U) were injected to prevent blood coagulation. Cardiac arrest was then achieved using 20 ml ice-cold Bretschneider solution (Custodiol®). The hearts were then excised after ligation of the venae cavae and the pulmonary veins and stored in cold lactated Ringers solution while the recipient rat was prepared. The recipient rat was anesthetized and prepared for surgery as described above and placed on a circulating-water heating pad. A ventral midline celiotomy was performed and the intestines were displaced to the right side and covered with gauze soaked with warm normal saline. The abdominal aorta and vena cava were isolated caudal to the kidneys and cranial to the aortic bifurcation and cross-clamped. An incision was made in both the aorta and vena cava and the vessels flushed with heparinized saline. The aorta of the donor heart was then sutured end to side to the recipient aorta in a simple continuous pattern with 8–0 nylon sutures, while the pulmonary artery of the donor heart was be sutured end to side with the vena cava. Following completion of the anastomosis, the clamps were removed from the aorta and vena cava (the caudal clamp above the aortic bifurcation is removed first, followed by the cranial clamp below the kidneys). The heart was then warmed with saline solution until it began to beat. The linea was then closed with absorbable suture material in a simple continuous pattern. Graft survival was monitored by daily palpation of the beating donor heart through the abdominal wall. The strength of the heartbeat was graded from weak ( =  1) to strong and brisk ( =  4). The time of rejection was defined as the last day of palpable cardiac contractions, and the rejection was confirmed by laparotomy. Postsurgical animals were further monitored for signs of pain, distress, dehydration, weight loss and general well-being. Monitoring occurred every 12 hours during the first day after surgery and then daily until graft failure. Buprenorphine was given at 5 µg/kg s.c. every 12 h for the first 24 h after surgery for pain control.

After transplantation animals received the following treatments once daily for 10 days and were then left untreated until the heterotopically transplanted hearts stopped beating: miglyol vehicle i.p., TRAM-34 i.p at 10 mg/kg or 40 mg/kg, TRAM-34 i.p. at 80 mg/kg (divided into two daily doses), sirolimus orally at 0.75 or 3 mg/kg, tacrolimus orally at 1, 2 or 8 mg/kg. Tacrolimus was obtained from Fujisawa GmbH (Munich, Germany) and was freshly dissolved in physiological saline. Sirolimus (Rapamune® oral solution) was from Wyeth (Münster, Germany). After the transplanted hearts were rejected, the animals were euthanized with an overdose of isoflurane. No animals died during the study period.

### Histopathology

Immersion fixed harvested aortas were paraffin embedded and prepared by the histopathology laboratory of the School of Veterinary Medicine at the University of California Davis. Sections of 5 µm thickness were cut from three levels of each harvested graft and stained with hematoxylin and eosin (H&E) to evaluate luminal occlusion and cellular infiltration. Briefly, sections stained with H&E were photographed and the resulting photos composited into whole-slide images and analyzed with Photoshop CS3 (Adobe systems incorporated). Adventitia, media and intima areas were outlined with the magnetic lasso tool, separated into different layers, the area determined in square micrometers (converted from pixel number obtained from the expanded histogram windows), and then averaged from three sections per graft. The proportion of vascular occlusion was calculated as follows [22]: Vascular occlusion (%)  =  [area of intima/(area of intima + vascular lumen)] ×100. Infiltration of mononuclear cells was evaluated according to the method of Lehr et al. [26]. Mononuclear cells were selected with the magic wand tool and counted in the different vessel areas.

### Immunohistochemistry

Sections were dewaxed with xylene, rehydrated through an alcohol gradient, and heated with 10 mM Na citrate (pH 6) in a microwave for 15 min to retrieve antigenic determinants. After treatment with 1% H_2_O_2_ to inactivate endogenous peroxidase activity and blocking with 5% goat serum in PBS, the sections were incubated overnight at 4°C with the primary antibody in PBS with 2% goat serum. The following primary antibodies were used: anti-KCa3.1 (1∶3000 for rat and 1∶1500 for human, AV35098, Sigma, MO), anti-rat CD68 (ED1, 1∶1000; Serotec, Raleigh, NC), anti-rat Kv1.3 (1∶750; P9107, Sigma), anti-human Kv1.3 (1∶500; ID8, Serotec, Raleigh, NC), α-smooth muscle actin (1∶200, Abcam, MA), and CD3 (6B10.2; 1∶200; Santa Cruz, CA). Bound primary antibodies were detected with a biotinylated donkey anti-mouse IgG secondary antibody (1∶500; Jackson ImmunoResearch, West Grove, PA) for CD68, CD3 and anti-human Kv1.3, or with biotinylated goat anti-rabbit IgG secondary antibodies (1∶500, Jackson ImmunoResearch, West Grove, PA) for anti-KCa3.1, anti-rat Kv1.3, and α-SMA followed by a horseradish peroxidase-conjugated avidin complex (Vectastain Elite ABC Kit, Vector Laboratories, Burlingame, CA). Peroxidase activity was visualized with 3,3'-diaminobenzidine (DAB Substrate Kit for Peroxidase, Vector Laboratories). Sections were counterstained with hematoxylin, dehydrated and mounted with Permount (both Fisher, Pittsburg, PA).

### Immunofluorescence

Sections were dewaxed with xylene, rehydrated through an alcohol gradient, and heated with 10 mM Na citrate (pH 6) in a microwave for 15 min to retrieve antigenic determinants. After blocking with 5% goat serum in PBS overnight, sections were incubated with the primary antibody in PBS with 2% goat serum at 4°C for 1 hour. The following primary antibodies were used: Rabbit anti-KCa3.1 (1 3000, AV35098, Sigma, MO), mouse anti-rat CD68 (1∶1000; ED1, Serotec), mouse anti-α smooth muscle actin (1∶800, Abcam), and mouse anti-CD43 (1∶1000; W3/13, Serotec). Bound primary antibodies were detected with an Alexa Fluor® 647-conjugated donkey anti-rabbit IgG secondary antibody (1∶500; Jackson ImmunoResearch, West Grove, PA) for KCa3.1, or with a FITC-conjugated goat anti-mouse IgG secondary antibody (1∶500, Jackson ImmunoResearch) for CD68, CD43 or α-SMA. Sections were mounted by Fluoromount-G (SouthernBiotech, Birmingham, AL) and imaged with a Zeiss LSM-510 confocal microscope.

### Specificity of KCa3.1 and Kv1.3 antibodies

The rabbit polyclonal Sigma AV35098 anti-KCa3.1 antibody, which according to its product description recognizes human and bovine KCa3.1, has previously been shown not to produce any unspecific staining in human brains or in the brains of wild-type or KCa3.1^−/−^ mice while it strongly stained human glioblastoma multiforme (see Supplementary Fig. 1 in [27]). We further previously used the Sigma AV35098 antibody (which should not be confused with the Sigma P4997 antibody, which produces a lot of unspecific staining in the brains of both wild-type and KCa3.1^−/−^ mice in our hands) to stain KCa3.1 on activated microglia following ischemic stroke in rats [28], and KCa3.1 on infiltrating T cells and macrophages in rat inflammatory bowel disease [29]. The specificity of the rabbit polyclonal Sigma anti-Kv1.3 antibody (P9107) is shown in Supporting Figure 2 in File S1 demonstrating staining on rat and mouse spleen but not spleen tissue from a Kv1.3^−/−^ mouse. The Serotec mouse monoclonal anti-human Kv1.3 antibody only recognizes human Kv1.3 and unlike the Sigma anti-Kv1.3 antibody does not stain wild-type mouse spleen in our hands. It also does not produce unspecific staining on Kv1.3^−/−^ mouse spleen (data not shown). KCa3.1^−/−^ mice were rederived and then bred by the Mouse Biology Program at the University of California, Davis. A formalin fixed spleen from a Kv1.3^−/−^ mouse was a generous gift from Leonard Kaczmarek at Yale University.

**Figure 1:**
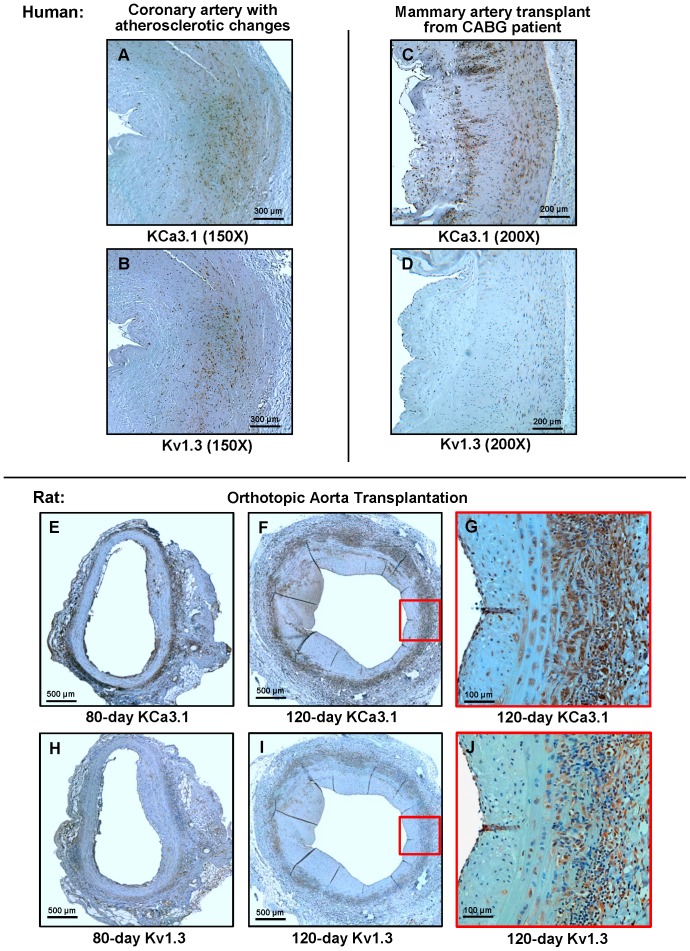
Kv1.3 and KCa3.1 expression in human and rat vasculopathy. (A, B) KCa3.1 and Kv1.3 staining in serial sections from a coronary artery with severe atherosclerotic changes. The vessel was harvested from the former heart of a patient receiving a heart transplant because of ischemic cardiomyopathy. (C, D) KCa3.1 and Kv1.3 staining in a mammary artery bypass graft. (E, F) KCa3.1 staining in orthotopic rat aorta transplants harvested 80 or 120 days after transplantation. G, Close-up of the boxed area in F. (H, I) Kv1.3 staining in serial sections of the grafts shown in E and F. (J) Close-up of the boxed area in F. Serial sections are 5 µm apart.

**Figure 2:**
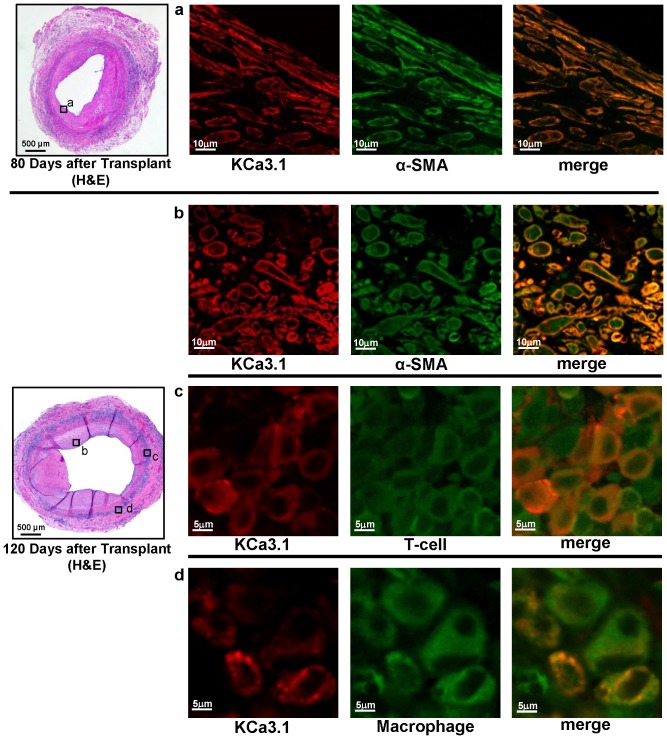
KCa3.1 staining localizes to smooth muscle cells, T cells and macrophages. Double fluorescent immunostaining for KCa3.1 and α-SMA, T cells (CD43) and macrophages (ED1, CD68) in orthotopic rat aorta transplants harvested 80 or 120 days after transplantation. The boxed areas in the H&E stained vessels on the right show the location where the fluorescent images were taken.

### Evaluation of collagen deposition

Collagen deposits were detected using Sirius red. Dewaxed and rehydrated aorta sections were stained for 1h in a solution containing 0.1% Sirius red (Pfaltz and Bauer, CT) and 0.5% picric acid. Sections were then washed twice with 100% ethanol, dehydrated and mounted with Permount. The sections were blinded and then evaluated by light microscopy. The deposition of collagen was scored from 0 to 5 (higher score meaning more deposition) as described [30].

### Plasma IFN-γ measurements

Plasma IFN-γ levels at time of sacrifice were determined with a rat IFN-γ ELISA set (BD OptEIATM, Cat. No. 558861) according to the manufacturer's protocol. Standards and plasma samples from each rat were run in duplicate and the results averaged.

### TRAM-34 and PAP-1 concentrations

TRAM-34 and PAP-1 plasma concentrations were determined using a Waters® ACQUITY UPLC stack (Waters Corporation, Milford, MA) coupled to an electrospray ionization TSQ↗Quantum Access MAX mass spectrometer (Thermo Fisher Scientific, Waltham, MA) as previously described [10], [28].

### Statistical analysis

Statistical analyses were performed using one-way ANOVA (Origin software). The Fisher Post Hoc test was used to compare the results of each group. The collagen deposition was analyzed by Mann-Whitney test. p<0.05 was used as the level of significance. *  =  p<0.05, **  =  p<0.01, ***  =  p<0.001.

## Results

### Kv1.3 and KCa3.1 expression in human and rat arteries with vasculopathy

High Kv1.3 expression has been previously reported on myelin and islet-antigen specific T cells from the blood of patients with multiple sclerosis and new onset type-1 diabetes [10] as well as on CD4^+^CD28^−^ T cells from patients with acute coronary syndrome [31], while KCa3.1 expression has been described in coronary vessels from patients with coronary artery disease and in atherosclerotic lesions in ApoE^−/−^ mice [21]. In order to determine if both channels are also present and therefore potentially involved in the pathogenesis of vasculopathy, we stained sections from a coronary artery from a patient with atherosclerotic changes and from a mammary artery coronary artery bypass graft (CABG), both exhibiting pronounced intimal hyperplasia, for Kv1.3 and KCa3.1 (Figure 1A-D). The coronary artery, which was harvested from the former heart of an ischemic cardiomyopathy patient prior to receiving a transplant, exhibited extensive KCa3.1 staining in the media and the more dense, α-smooth muscle actin-positive area of the neointima, but only very scattered staining, predominantly localized to a few infiltrating lymphocytes in the loose subendothelial area of the neointima. Similarly, Kv1.3 staining was more pronounced in the denser part of the neointima but overall showed a more scattered appearance than the KCa3.1 staining and was mostly absent from the media. The mammary artery from the CABG patient, in contrast, exhibited no significant Kv1.3 staining in keeping with the absence of any significant memory T cell reactivity, but showed intense KCa3.1 staining in the media as well as on accumulations of mononuclear cells in the neointima and on more scattered cells in the looser part of the neointima facing the lumen.

A similar KCa3.1 and Kv1.3 staining pattern was observed in a rat aorta transplant model in which a piece of the thoracic aorta from PVG rats was orthotopically transplanted into the infrarenal abdominal aorta of ACI rats by end to end anastomoses [23]. This model develops chronic vasculopathy with typically about 20% luminal occlusion 80 days after transplantation and roughly 50% after 120 days [22]. After both 80 and 120 days, transplants exhibited KCa3.1 staining in the media and on infiltrating mononuclear cells in the adventitia, forming a ring around the vessel (1E and 1F), and on occasional clusters of mononuclear cells in the neointima and below the endothelium (1E). As shown on the serial sections in Supporting Figure 1 in File S1, the KCa3.1 staining in allografts was generally localized to both smooth muscle cell and ED1^+^ macrophage containing areas, while isografts exhibited the most intense KCa3.1 staining in the vascular endothelium where the channel is known to be expressed and where it participates in endothelium derived hyperpolarization [32]. Kv1.3 staining in contrast was much less intense and primarily localized to areas of mononuclear cell infiltration (1H – 1J).

Higher magnification of double fluorescent immunostaining revealed localization of KCa3.1 immunoreactivity to α-SMA^+^ cells in the neointima and to adventitia infiltrating T cells and macrophages (Figure 2). In keeping with the continuous remodeling in the expanding neointima, subendothelial smooth muscle cells on day-80 after transplantation were predominantly spindly shaped while smooth muscle cells in the thicker initima on day-120 after transplantation exhibited various shapes including epithelioid and more rhomboid and appeared less ordered (Figure 2).

### KCa3.1 blockade reduces luminal occlusion and intimal hyperplasia

**Figure 3:**
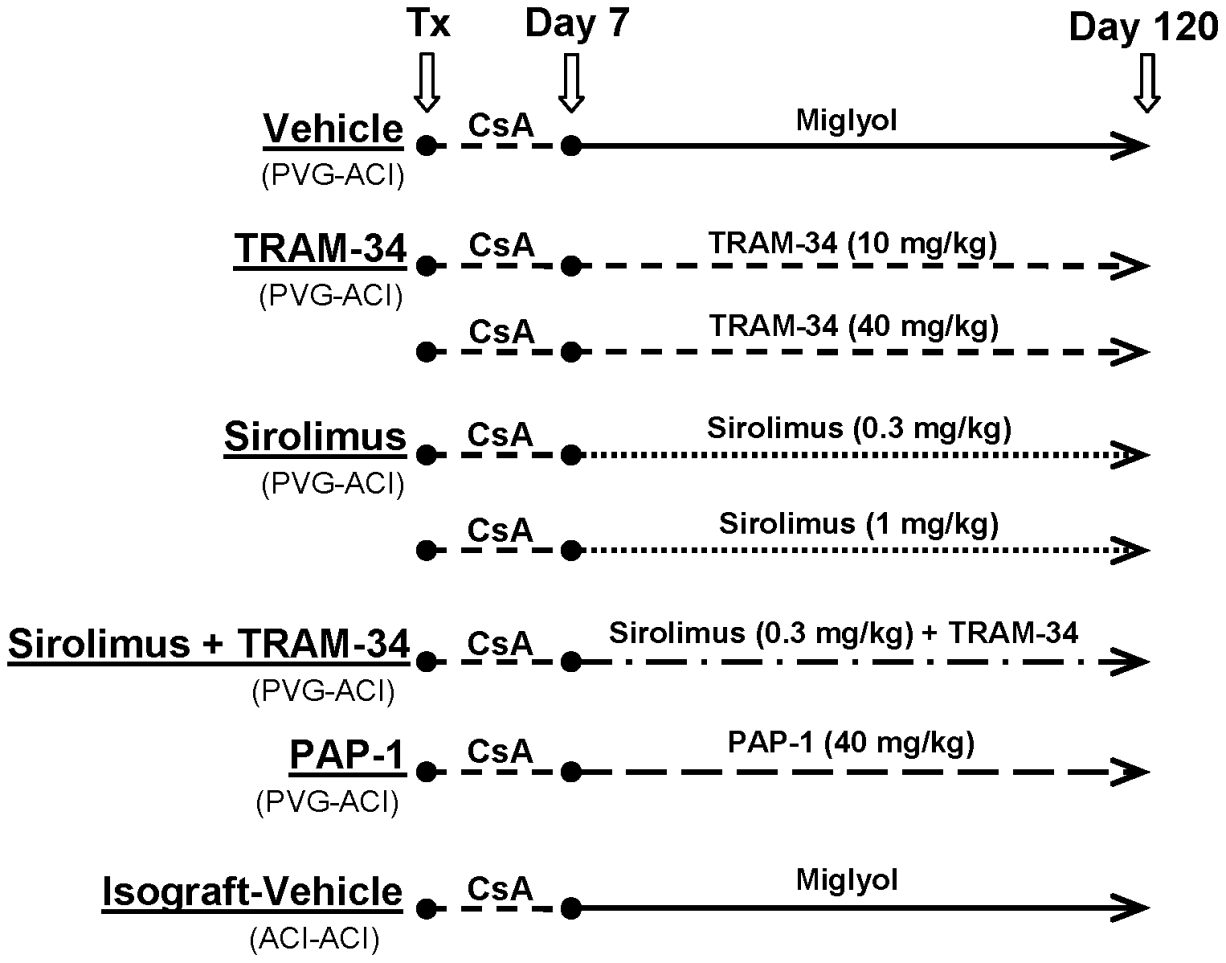
Treatment scheme.

Since immunohistochemistry had demonstrated the presence of Kv1.3 primarily on infiltrating T cells and the expression of KCa3.1 on macrophages, some T cells and on neointimal smooth muscle cells we evaluated the efficacy of the KCa3.1 blocker TRAM-34 and the Kv1.3 blocker PAP-1 in the PVG-ACI aortic transplant model according to the treatment scheme in Figure 3. Following a brief 7-day course of cyclosporine A to prevent acute rejection and achieve long-term survival of the graft and development of chronic AV, rats were treated either with vehicle, low or high dose TRAM-34, PAP-1, or low and high dose sirolimus (rapamycin) for another 113 days. We further included a group treated with a combination of low dose TRAM-34 and low dose sirolimus to study potential synergy and an isograft group where aortas were transplanted from ACI to ACI rats. The respective doses of TRAM-34 (10 and 40 mg/kg) and PAP-1 (40 mg/kg) were chosen since these doses had previously been used by us in other studies, where they had been well tolerated and achieved pharmacologically active plasma concentrations [10], [28].

**Figure 4:**
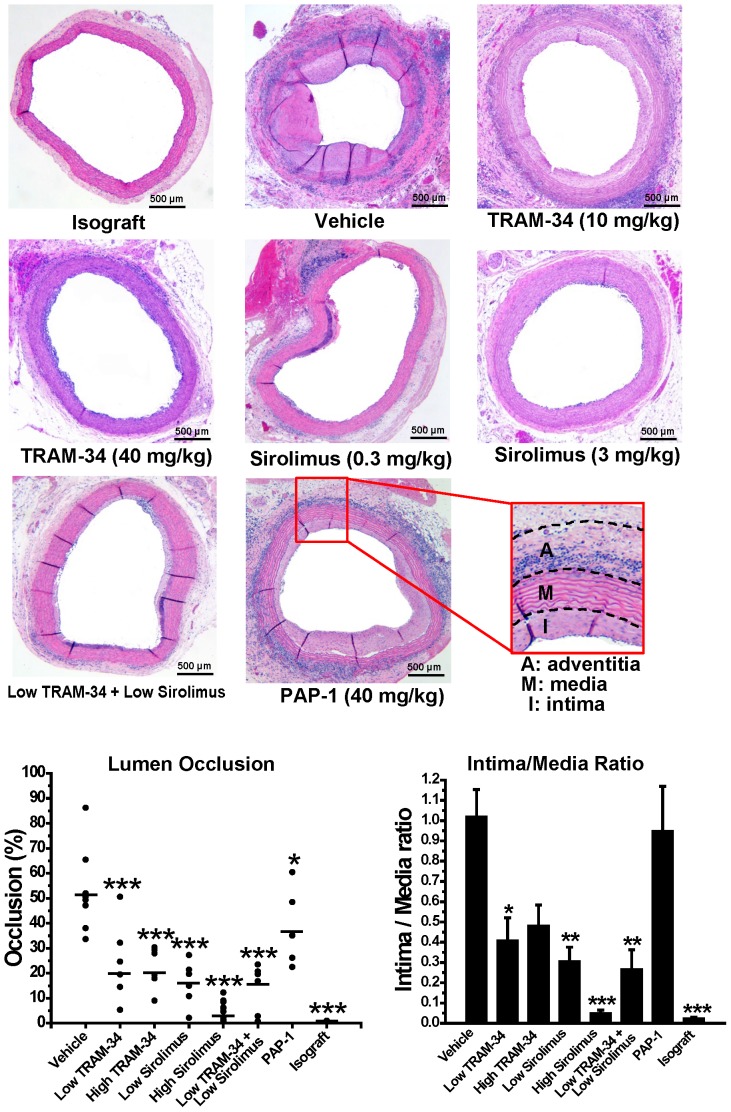
KCa3.1 blockade reduces luminal occlusion. (A) Representative images of H&E stained aortic grafts harvested 120 days after transplantation (50-fold magnification). (B) Percentage of luminal occlusion and intima/media ratios evaluated at three different levels of each harvested graft in the different treatment groups. For averaged values and statistics see Supporting Table 1 in File S1.

Similar to what has been previously described for the PVG-ACI model, grafts harvested from vehicle treated animals 120 days after transplantation exhibited an occlusion percentage of 52.8±5.8% (n = 8) of the lumen (Figure 4). Treatment with low (10 mg/kg) or high (40 mg/kg) doses of the KCa3.1 blocker TRAM-34 resulted in a significant reduction of luminal occlusion (21.5±6.16% for low dose, n = 7; and 22.4±3.42% for high dose, n = 6) and intimal proliferation (see media/intima ratio in Fig. 4). The effects of TRAM-34 were comparable to low dose sirolimus (16.1±3.6% occlusion, n = 6), but not as pronounced as with high-dose sirolimus (3.87±1.2% occlusion, n = 6). The combination of low dose sirolimus with low dose TRAM-34 (16.7±3.7% occlusion, n = 5) was not more effective than low dose sirolimus alone. Treatment with the Kv1.3 blocker PAP-1 (37.9±5.8% occlusion, n = 6) had a small effect on luminal occlusion but in contrast to TRAM-34 or sirolimus treatment did not reduce the intima/media ratio.

**Figure 5:**
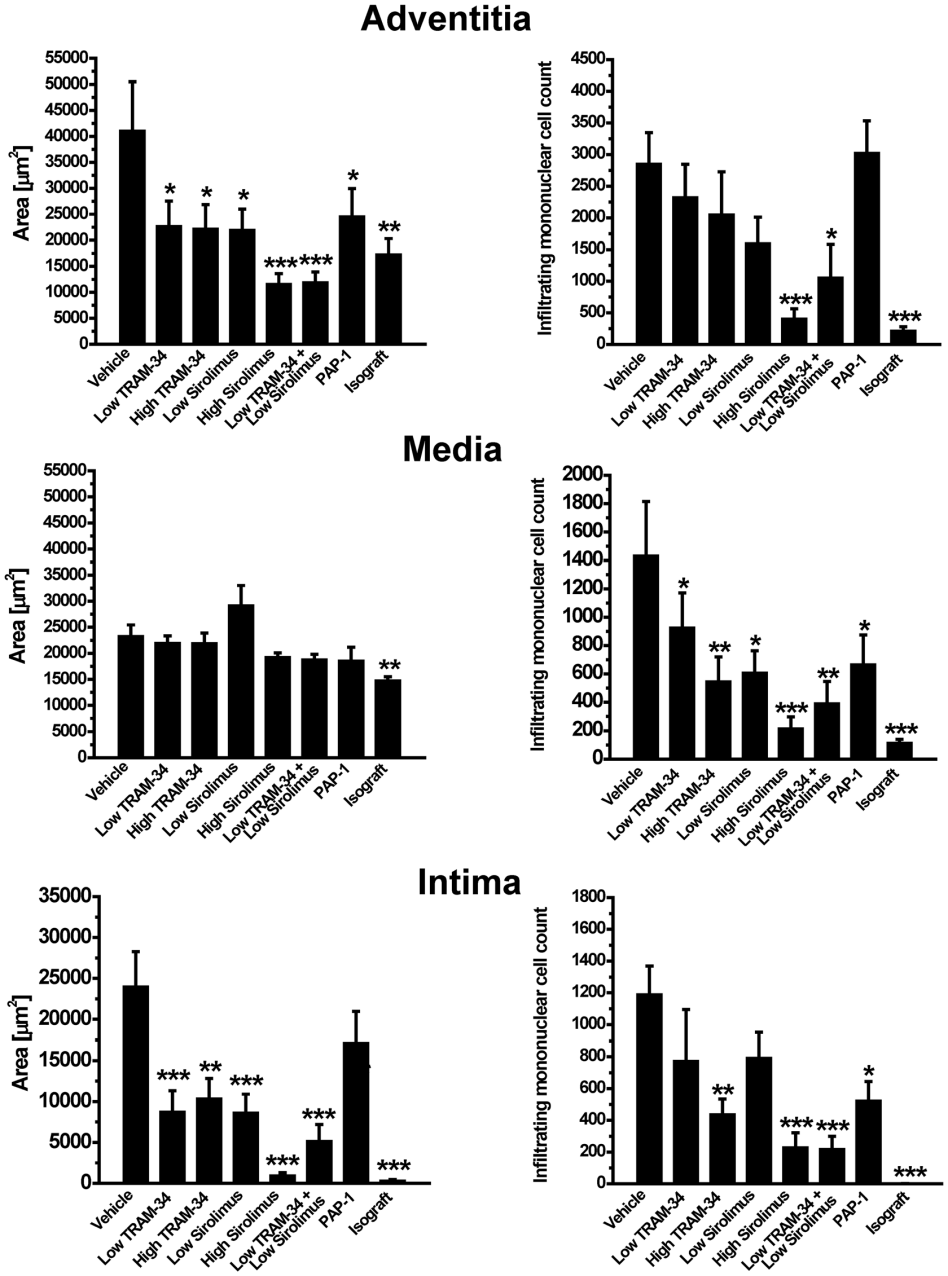
Effects of treatment on adventitia, media and intima areas and mononuclear cell infiltration. Averaged adventitia, media and intima areas measured at three levels in each harvested graft are shown inµm^2^. Total mononuclear cell numbers were determined in the same sections. For averaged area values, total numbers of infiltrating cells and statistics see Supporting Table 2 in File S1.

A more detailed analysis of areas and total numbers of infiltrating mononuclear cells in adventitia, media and intima of the harvested vessel grafts (Figure 5) revealed that while there was no change in media area, all treatments with the exception of PAP-1 significantly reduced intima formation on day-120. Both the low and the high doses of the KCa3.1 blocker TRAM-34 were as effective at reducing intima formation as low dose sirolimus, but less effective than high dose sirolimus. Interestingly, only the high doses of TRAM-34 and sirolimus as well as the combination of low dose TRAM-34 and low dose sirolimus significantly reduced mononuclear cell infiltration in the media and intima demonstrating, that while low doses of both agents are able to inhibit smooth muscle cell proliferation, higher doses are necessary to significantly affect immune cell infiltration (Figure 5). The Kv1.3 blocker PAP-1, in contrast, only reduced infiltration in media and intima but did not show a significant effect of intimal growth. Adventitia area increases, which are primarily due to intense immune cell infiltration, were drastically reduced by high dose sirolimus and the combination of low dose TRAM-34 and low dose sirolimus and to a lesser degree by the other treatments.

### PAP-1, high dose sirolimus and the combination of low dose sirolimus and low dose TRAM-34 strongly reduce circulating IFN-γ levels

**Figure 6:**
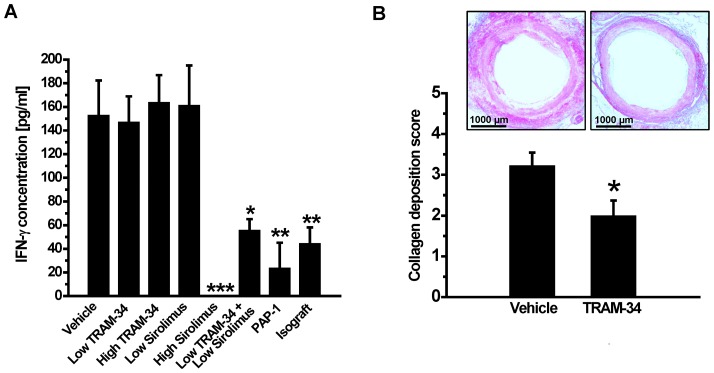
(A) PAP-1, high dose sirolimus and the combination of low dose sirolimus and low dose TRAM-34 strongly reduce circulating IFN-γ levels. IFN-γ levels in pg/ml on day-120 after transplantation: Vehicle (153.0±29, n = 8); low-dose TRAM-34 (147.3±21.6, n = 7); high-dose TRAM-34 (163.89±23, n = 6); low-dose sirolimus (161.4±33.7, n = 6); high dose sirolimus (not detectable, n = 5); low-dose TRAM-34 plus low-dose sirolimus (55.7±9.4, n = 6, p = 0.01), PAP-1 (23.8±21.3, n = 6, p = 0.001); isograft (44.6±13.6, n = 6, p = 0.006). (B) TRAM-34 reduces collagen deposition. Representative images of Sirius red stained aortic grafts harvested 120 days after transplantation from vehicle and TRAM-34 treated rats and collagen deposition score from of aortic grafts from vehicle (3.22±0.32, n = 8) and high-dose TRAM-34 treated rats (2±0.37, n = 6, p = 0.045).

The classical Th1 cytokine IFN-γ plays a crucial role in the development of AV based on the observations that both genetic knockout or neutralization of IFN-γ with a monoclonal antibody prevent AV in mice [1]. We therefore measured plasma IFN-γ levels in all groups on day-120. While rats treated with low or high dose TRAM-34 or low dose sirolimus exhibited similar circulating IFN-γ amounts as vehicle treated animals (∼150 pg/ml), the combination of low dose TRAM-34 with low dose sirolimus significantly reduced IFN-γ levels suggesting a synergistic effect between KCa3.1 blockade and mTOR inhibition (Figure 6). IFN-γ levels were also drastically suppressed by high dose sirolimus and by the Kv1.3 inhibitor PAP-1 (Figure 6). Both treatments reduced IFN-γ levels below basal level of around 80 pg/ml in rats.

### KCa3.1 blockade reduces collagen deposition

Since KCa3.1 blockade or genetic deletion of the channel has previously been reported to inhibit fibroblast proliferation and to reduce interstitial collagen deposition in a model of renal fibrosis [20], we postulated that KCa3.1 blockade might also be able to reduce the collagen deposition and often constrictive adventitial and media fibrosis that typically accompanies chronic AV [1]. Sirius red staining revealed that grafts harvested on day-120 from TRAM-34 treated rats indeed exhibited reduced collagen deposition significantly (score: 2.00±0.37) compared to vehicle treated grafts (score: 3.22±0.32) (Figure 6).

### KCa3.1 blockade does not prevent acute allograft rejection

**Figure 7:**
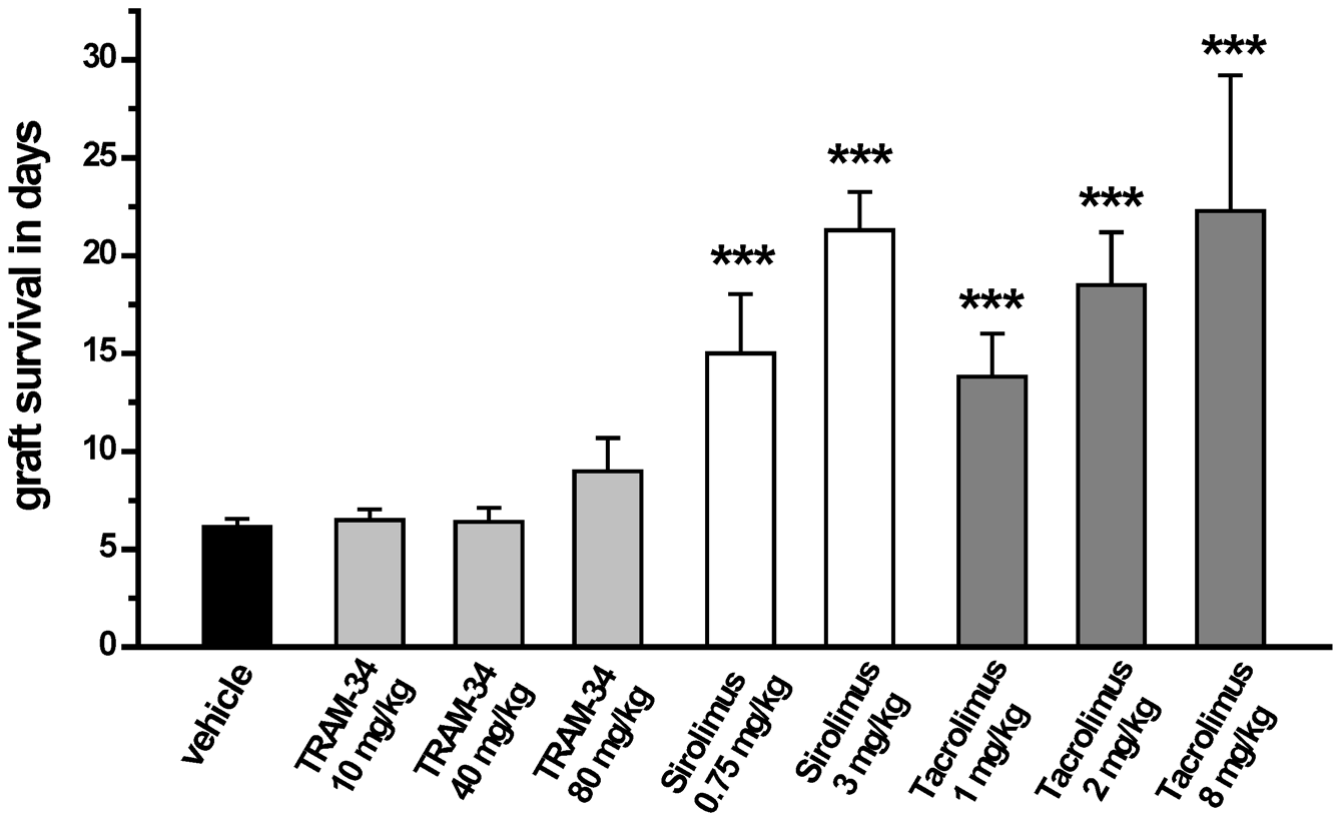
Graft survival times of heterotopic BN to Lewis heart transplants after a 10-day treatment period. Grafts were monitored by abdominal palpitation and defined as rejected when they reached a beating score of 0. Graft survival times in days: vehicle 6.2±0.4; TRAM-34 10 mg/kg 6.5±0.5; TRAM-34 40 mg/kg 6.4±0.7; TRAM-34 80 mg/kg 9±1.4; sirolimus 0.75 mg/kg 15±3.3; sirolimus 3 mg/kg 21.3±1.9; tacrolimus 1 mg/kg 13.8±2.2; tacrolimus 2 mg/kg 18.5±2.7; tacrolimus 8 mg/kg 22.3±6.9.

Based on the fact that TRAM-34, which has been reported to inhibit mitogen stimulated human and rat T cell proliferation with IC_50_s in the range of 250 nM to 1 µM [16], [29], [33], also suppresses [^3^H]-thymidine incorporation in a one-way mixed lymphocyte reaction (MLR) between Lewis rat splenocytes and irradiated Brown Norway (BN) splenocytes with an IC_50_ of 250 nM (data not shown), we tested TRAM-34 in a rat model of acute rejection. Hearts were heterotopically transplanted from BN to Lewis rats and recipients then treated for 10 days with various doses of TRAM-34, sirolimus or tacrolimus (Figure 7). This fully mismatched heterotopic heart transplant model is very well established and often used to investigate the potency of new immunosuppressives to achieve long-term allograft survival [34]–[36].

In contrast to its efficacy in the vasculopathy model, TRAM-34, administered at 10 or 40 mg/kg (6.4±0.7 days), did not prolong graft survival in this acute model when compared to untreated animals (6.2±0.4 days). A higher dose of 80 mg/kg administered in two 40 mg/kg doses showed a slight trend towards prolonged survival (9±1.4 days), however, all animals still rejected before day-10. In contrast, grafts from animals treated with high doses of the mTOR inhibitor sirolimus (3 mg/kg) or the calcineurin inhibitor tacrolimus (8 mg/kg), survived for 22 days (Figure 7), which is 12 days after drug treatment was discontinued. Lower doses of both compounds showed less pronounced effects (Figure 7).

## Discussion

A recent retrospective study of 64 explanted hearts from adult and pediatric patients undergoing retransplantation revealed lesions demonstrating concentric fibromuscular intimal hyperplasia as the most prominent pathology affecting large and small arteries, as well as veins [3]. However, many lesions also contained inflammatory infiltrates consisting of CD3^+^ T cells and CD68^+^ macrophages and/or demonstrated features of atherosclerosis, even in pediatric patients, suggesting that chronic AV can be defined as a pathological triad of fibromuscular intimal hyperplasia, vasculitis and atherosclerosis. Taken together with a large body of earlier work (see [1] for a review), these findings suggest that therapies for AV should ideally target all three pathologies. We here found a similar pathology involving fibromuscular initimal hyperplasia and vasculitis with macrophage and T cell infiltration in a rat AV model and demonstrate that TRAM-34, a blocker of the Ca^2+^-activated K^+^ channel KCa3.1 prevents AV development.

Since KCa3.1 is expressed in T cells, macrophages, dedifferentiated vascular smooth muscle cells and fibroblasts, all of which are involved in AV pathogenesis, TRAM-34 probably targets proliferation and activation processes in all these cells in our rat AV model. However, the fact that low dose TRAM-34 reduced area increases in the adventitia and the intima as effectively as low dose sirolimus without significantly affecting mononuclear cell infiltration suggests that the most sensitive target for KCa3.1 blockers in AV are proliferating smooth muscle cells and fibroblasts. As first reported by Neylon et al. in 1999 [37], the switch of vascular smooth muscle cells from a contractile to a proliferative phenotype is accompanied by a change in K^+^ channel expression from the large conductance BK (KCa1.1) channel to the intermediate-conductance KCa3.1 channel. This observation was later confirmed *in vivo* in restenosis models in both rats [18] and pigs [19] and has been shown to be accompanied by a move of the repressor element 1-silencing transcription factor (REST), which typically keeps the KCa3.1 (KCNN4) gene repressed, out of the nucleus resulting in increased KCa3.1 message and protein expression [19], [38]. In this phenotypic switch, increased KCa3.1 expression is part of a major change in Ca^2+^ handling mechanisms. As KCa1.1 and L-type Ca^2+^ channels (Cav1.2) are down-regulated, TRPCs, IP_3_ receptors, STIM1 and Orail1 expression increases [39], [40], so that Ca^2+^ entry in proliferating vascular smooth muscle cells is now triggered by hyperpolarization instead of depolarization [41]. Activated by increases in intracellular Ca^2+^, KCa3.1 maintains a negative membrane potential and further facilitates Ca^2+^ entry and proliferation. TRAM-34 has accordingly been reported to inhibit VSMC proliferation with IC_50_s in the range of 8 to 100 nM by reducing Ca^2+^ influx and arresting cell cycle in the G_0_/G_i_ phase [18], [21], [38], [42]. Similar findings have been reported in fibroblasts [20], [43]. The high sensitivity of VSMC to KCa3.1 blockers is in line with our observation that even 10 mg/kg TRAM-34, which results in plasma trough levels of 109±19 nM (n = 7), significantly reduced intimal hyperplasia. The higher TRAM-34 dose of 40 mg/kg, which resulted in plasma trough levels of 748±430 nM (n = 6), additionally inhibited mononuclear cell infiltration in the media and intima in keeping with TRAM-34’s reported T cell and macrophage suppressive effects at concentrations of 250 nM to 1 µM [16], [21], [29], [33]. Similar TRAM-34 plasma concentrations have also previously been found to prevent atherosclerosis development in ApoE^−/−^ mice through a combination of suppressing VSMC proliferation and decreasing infiltration of macrophages and T cells into atherosclerotic plaques [21] supporting our assumption that higher concentrations of KCa3.1 inhibitors “hit” both the fibroproliferative and the inflammatory component of AV.

The Kv1.3 blocker PAP-1 in contrast did not significantly reduce intimal hyperplasia despite reducing mononuclear cell infiltration and drastically reducing plasma IFN-γ levels suggesting that, at least in the rat AV model, VSMC proliferation contributes more significantly to AV pathology than CCR7^−^ effector memory T cells. The lack of effect of the Kv1.3 blocker is probably not due to insufficient exposure since PAP- plasma trough levels in our study averaged 1.157±0.541 µM (n = 5) and the same PAP-1 dose effectively prevents autoimmune diabetes development in diabetes-prone BB/W rats [10]. Our findings further suggest that Kv1.3 is not involved in VSMC proliferation in our rat AV model as has been suggested by a study using cultured VSMC [44] and a high-throughput real-time polymerase chain reaction of 87 ion channels performed on injured mouse femoral arteries and cultured VSMC [45]. The latter study identified Kv1.3 and the Kvβ2 as the only two genes increasing in expression during proliferation and failed to identify KCa3.1, a finding that has been suggested to be either due to species differences and different culture conditions or to the lack of fidelity between mRNA and functional protein expression [46].

Based on our study we would like to suggest KCa3.1 blockers like the TRAM-34 analog Senicapoc as potential novel therapeutics to prevent AV and thus eventual ischemic graft failure of transplanted hearts or kidneys. Senicapoc (a.k.a. PF-05416266 or ICA-17043) was recently deposited in the NCATS (National Center for Advancing Translational Sciences) library and would be available for investigator initiated clinical trials. Similar to our academic tool compound TRAM-34, which exhibits an excellent selectivity over other ion channels and did not induce any toxicity in a 28-day toxicity study in mice and in a 6-months toxicity study in rats [28], Senicapoc was safe and well tolerate in a Phase-1 clinical trial in healthy volunteers [47], and was afterwards found to not induce any adverse events in over 500 sickle cell anemia patients taking Senicapoc for up to two years in Phase-2 and Phase-3 clinical trials [48]. Triarylmethane-type KCa3.1 blockers accordingly seem to be relatively safe, although the potential of toxicity arising through drug-drug interactions during combination therapy cannot be excluded, and Senicapoc could be added to existing transplantation treatment regiments in patients at risk for developing or already exhibiting AV. KCa3.1 inhibitors would of course have to be used in combination with other immunosuppressants since they primarily affect the fibroproliferative component of AV and are not strong enough immunosuppressants to effectively prevent rejection in the setting of a strong MHC mismatch as demonstrated by our heterotopic heart transplant experiments. However, in this context it should be mentioned that TRAM-34 was recently reported to prevent airway obliteration in a mouse trachea transplant model by reducing T cell and myofibroblast activity [49] and had been previously found to inhibit T cell and macrophage infiltration in a Fisher-Lewis rat kidney transplant model in combination with the Kv1.3 blocker ShK [50]. TRAM-34 was further effective in a mouse model of inflammatory bowel disease [51], in kidney fibrosis [20] and most recently in a mouse asthma model where it prevented bronchial smooth muscle remodeling and sub-basement collagen deposition [52]. However, TRAM-34 did not delay influenza virus clearance in rats [21] and sickle cell anemia patients taking Senicapoc did not exhibit any increases in the number of infections [48]. So overall, KCa3.1 blockers seem to be relatively mild immunosuppressants, which would be ideally suited for targeting the fibroproliferative component of AV and other diseases in combination with other, stronger immunosuppressants. In this setting, we would like to suggest that KCa3.1 blockers could be used to reduce sirolimus or cyclosporine doses and help to reduce the side-effects of these agents. We tried to test for synergism between sirolimus and TRAM-34 by combining low doses of both agents in our study and while we could show a clear advantage of the combination on plasma IFN-γ and mononuclear cell infiltration in the adventitia and the media, both compounds were so effective at reducing intimal hyperplasia in our rat model that we failed to detect a clear synergism.

In conclusion, our present study suggests that KCa3.1 channels play an important role in the pathogenesis of chronic AV and we would like to suggest that KCa3.1 blockers like Senicapoc constitute promising therapeutics for the prevention of arteriopathy in transplanted organs.

## Supporting Information

File S1 Supporting Table 1. Percentage of luminal occlusion and intima/media ratios evaluated at three different levels of each harvested graft in the different treatment groups. Supporting Table 2. Averaged adventitia, media and intima areas measured at three levels in each harvested graft are given in µm^2^. Total mononuclear cell numbers were determined in the same sections. Supporting Figure 1. KCa3.1 expression in rat vasculopathy. (A-D) Serial sections stained with H&E or antibodies against α-SMA, the macrophage marker ED1 ( =  CD68) and KCa3.1. Sections A, B, C and D are sequential and each section is 5 µm from the previous section. (E) KCa3.1 expression on the vascular endothelium of an isograft. (F) Close-up of the boxed area in E. Supporting Figure 2. Specificity of Kv1.3 antibody (Sigma P9170) in mouse and rat spleen. (A, B) Sequential rat spleen sections stained with the polyclonal anti-Kv1.3 antibody (P9170; 1:750) or secondary antibody only. (C, D, E) Kv1.3 staining in mouse spleen. C and D are wild-type mouse spleen sections stained with anti-Kv1.3 (1∶750) or secondary antibody only. E shows a Kv1.3 knock-out mouse spleen section stained for Kv1.3 (1∶750). In all sections secondary antibody binding is visualized by DAB. Sections are counterstained with hematoxylin.
(PDF)
