# Supplementary material for: The Ca2+-Activated K+ Channel KCa3.1 as a Potential New Target for the Prevention of Allograft Vasculopathy
Source: PLoS One. 2013 Nov 29;8(11):e81006. doi: 10.1371/journal.pone.0081006 (PMC3843675; doi:10.1371/journal.pone.0081006)
Supplement: File S1 — Supporting Table 1. Percentage of luminal occlusion and intima/media ratios evaluated at three different levels of each harvested graft in the different treatment groups. Supporting Table 2. Averaged adventitia, media and intima areas measured at three levels in each harvested graft are given in µm2. Total mononuclear cell numbers were determined in the same sections. Supporting Figure 1. KCa3.1 expression in rat vasculopathy. (A-D) Serial sections stained with H&E or antibodies against α-SMA, the macrophage marker ED1 ( = CD68) and KCa3.1. Sections A, B, C and D are sequential and each section is 5 µm from the previous section. (E) KCa3.1 expression on the vascular endothelium of an isograft. (F) Close-up of the boxed area in E. Supporting Figure 2. Specificity of Kv1.3 antibody (Sigma P9170) in mouse and rat spleen. (A, B) Sequential rat spleen sections stained with the polyclonal anti-Kv1.3 antibody (P9170; 1:750) or secondary antibody only. (C, D, E) Kv1.3 staining in mouse spleen. C and D are wild-type mouse spleen sections stained with anti-Kv1.3 (1∶750) or secondary antibody only. E shows a Kv1.3 knock-out mouse spleen section stained for Kv1.3 (1∶750). In all sections secondary antibody binding is visualized by DAB. Sections are counterstained with hematoxylin. (PDF) [file pone.0081006.s001.pdf]

Treatment group	Luminal occlusion	Intima/media ratio
Vehicle (n = 8)	52.8 ± 5.8%	1.02 ± 0.13
low-dose TRAM-34 (n = 7)	21.5 ± 6.2% ($p = 1.65x10^{-5}$)	0.41 ± 0.11 ($p = 4.87x10^{-4}$)
high-dose TRAM-34 (n=6)	22.4 ± 3.4% ($p = 4.72x10^{-5}$)	0.49 ± 0.10 (p = 0.003)
low-dose sirolimus (n = 6)	16.1 ± 3.6% ($p = 2.17x10^{-6}$)	0.31 ± 0.07 ($p = 1.25x10^{-4}$)
high-dose sirolimus (n = 6)	3.9 ± 1.2% ($p = 4.89x10^{-9}$)	0.05 ± 0.01 ($p = 8.64x10^{-7}$)
low-dose TRAM-34 plus low-dose sirolimus (n = 5)	16.7 ± 3.7% ($p = 7.66x10^{-6}$)	0.27 ± 0.09 ($p = 1.24x10^{-4}$)
PAP-1 (n = 6)	38.0 ± 5.8% (p = 0.03)	0.95 ± 0.22
isograft (n = 4)	0.6 ± 0.2% ($p = 2.08x10^{-8}$)	0.03 ± 0.00 ($p = 5.3x10^{-6}$)

Supporting Table 1.
Percentage of luminal occlusion and intima/media ratios evaluated at three different levels of each harvested graft in the different treatment groups.

Treatment group	Adventitia [μm]	Adventitia MNCs	Media [μm]	Media MNCs	Intima [μm]	Intima MNCs
Vehicle (n = 8)	41248 ± 9242	2865 ± 480	23462 ± 1992<br>p = NS	1439 ± 375	24090 ± 4185	1195 ± 173
low-dose TRAM-34<br>(n = 7)	22856 ± 4686<br>p = 0.01	2336 ± 511<br>p = NS	22145 ± 12078<br>p = NS	932 ± 240<br>p = 0.09	8832 ± 2488<br>$p = 3.6x10^{-4}$	778 ± 317<br>p = NS
high-dose TRAM-34<br>(n=6)	22369 ± 4474<br>p = 0.01	2063 ± 665<br>p = NS	22060 ± 1830<br>p = NS	551 ± 169<br>p = 0.006	10473 ± 2359<br>p = 0.002	442 ± 92<br>p = 0.002
low-dose sirolimus<br>(n = 6)	22101 ± 3897<br>p = 0.01	1610 ± 402<br>p = NS	29347 ± 3684<br>p = NS	613 ± 150<br>p = 0.01	8718 ± 2180<br>$p = 5.40x10^{-4}$	799 ± 154<br>p = 0.09
high-dose sirolimus<br>(n = 6)	4612 ± 1883<br>$p = 2.1x10^{-4}$	417 ± 147<br>$p = 3.89x10^{-4}$	19423 ± 659<br>p = NS	221 ± 74<br>$p = 2.88x10^{-4}$	1051 ± 243<br>$p = 1.32x10^{-6}$	233 ± 89<br>$p = 1.16x10^{-4}$
low-dose TRAM-34 plus low-dose sirolimus<br>(n = 5)	12045 ± 1887<br>$p = 4.55x10^{-4}$	1069 ± 516<br>p = 0.01	18970 ± 834<br>p = NS	396 ± 149<br>p = 0.003	5255 ± 1930<br>$p=8.4x10^{-5}$	224 ± 74<br>$p = 2.08x10^{-4}$
PAP-1 (n = 6)	24691 ± 5267<br>p = 0.03	3039 ± 493<br>p = NS	18697 ± 2464<br>p = NS	670 ± 203<br>p = 0.017	17261 ± 3712<br>p = 0.10	528 ± 117<br>p = 0.005
isograft (n = 4)	17442 ± 2877<br>p = 0.006	229 ± 51<br>$p = 6.96x10^{-4}$	14893 ± 604<br>p = 0.0058	119 ± 21<br>$p = 4.89x10^{-4}$	410 ± 45<br>$p = 7.6x10^{-6}$	1.75 ± 0.25<br>$p=3.26x10^{-5}$

Supporting Table 2.
Averaged adventitia, media and intima areas measured at three levels in each harvested graft are given in μm^2. Total mononuclear cell numbers were determined in the same sections.

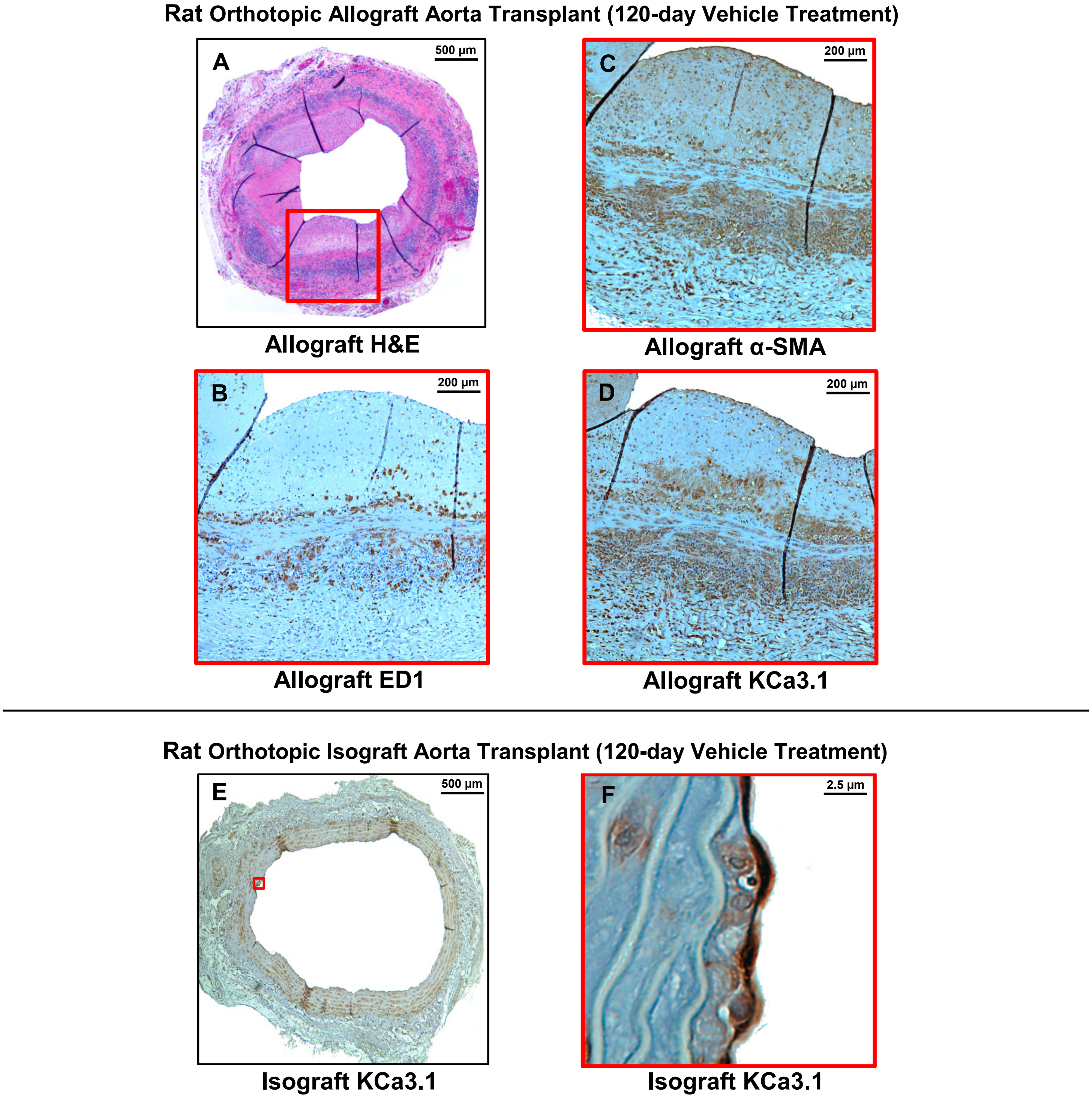

Supporting Figure 1. KCa3.1 expression in rat vasculopathy. A, B, C, D, Serial sections stained with H&E or antibodies against α-SMA, the macrophage marker ED1 (= CD68) and KCa3.1. Sections A, B, C and D are sequential and each section is 5 µm from the previous section. **E**, KCa3.1 expression on the vascular endothelium of an isograft. **F,** Close-up of the boxed area in E.

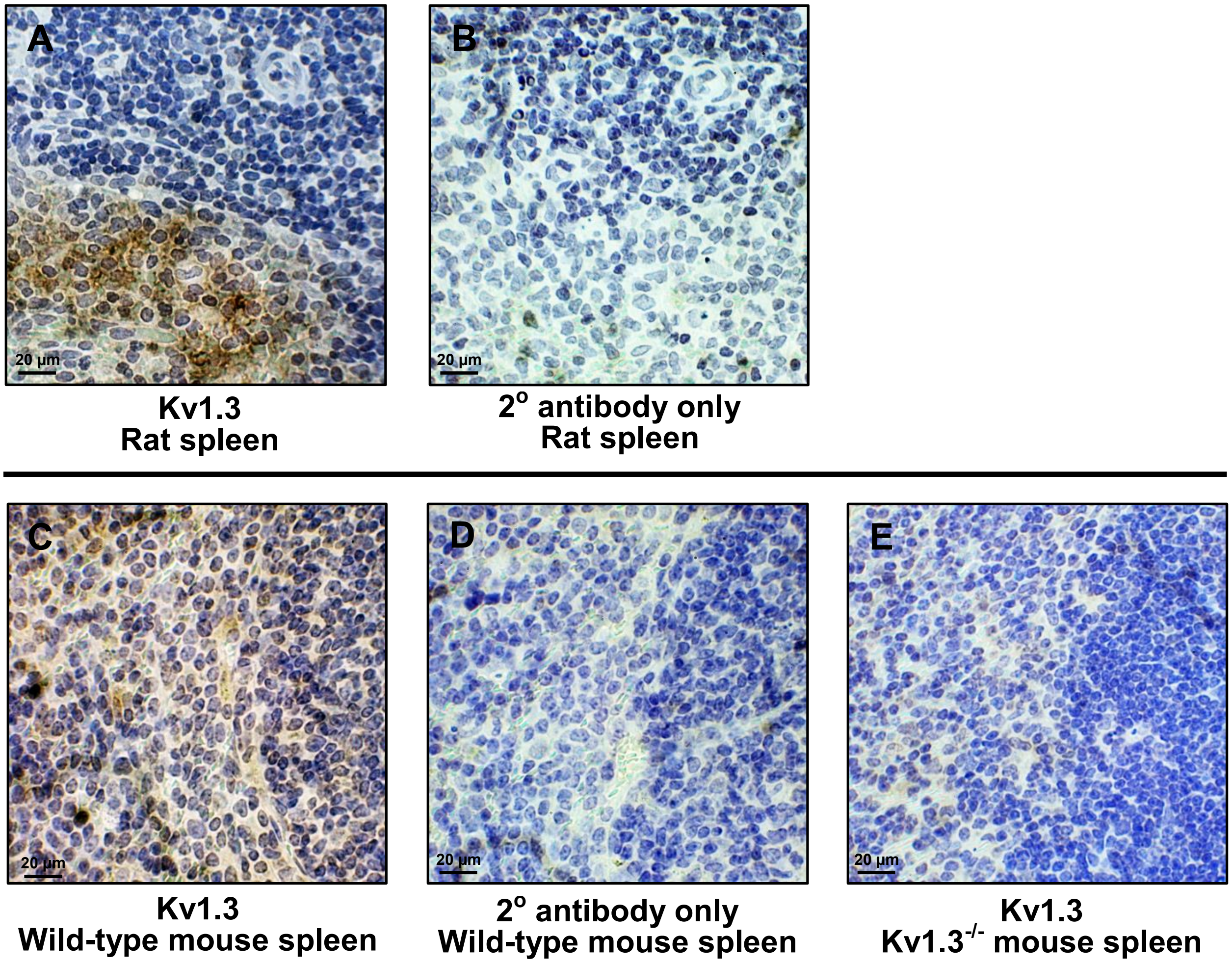

Supporting Figure 2. Specificity of Kv1.3 antibody (Sigma P9170) in mouse and rat spleen.
A, B, Sequential rat spleen sections stained with the polyclonal anti-Kv1.3 antibody (P9170; 1:750) or secondary antibody only. **C, D, E,** Kv1.3 staining in mouse spleen. C and D are wild-type mouse spleen sections stained with anti-Kv1.3 (1:750) or secondary antibody only. E shows a Kv1.3 knock-out mouse spleen section stained for Kv1.3 (1:750). In all sections secondary antibody binding is visualized by DAB. Sections are counterstained with hematoxylin.
